# Applying the intervention Complexity Assessment Tool to brief interventions targeting long-term benzodiazepine receptor agonist use in primary care: Lessons learned

**DOI:** 10.1186/s12875-022-01775-y

**Published:** 2022-07-16

**Authors:** Aisling Barry, Simon Lewin, Cathal A. Cadogan

**Affiliations:** 1 School of Pharmacy and Pharmaceutical Sciences, Trinity College Dublin, Dublin, Ireland; 2 Division of Health Services, Norwegian Institute of Public Health, Oslo, Norway; 3 Health Systems Research Unit, South African Medical Research Council, Cape Town, South Africa; 4 Department of Health Sciences, Norwegian University of Science and Technology, Ålesund, Norway

**Keywords:** Benzodiazepines, Z-drugs, Brief interventions, Intervention complexity, Behaviour change techniques, Systematic review, Intervention complexity, iCAT_SR

## Abstract

**Background:**

Benzodiazepine receptor agonists (BZRAs) are often prescribed for long-term use. However, guidelines recommend limiting prescriptions to short-term use (< 4 weeks) to reduce the risk of adverse effects and dependence. A recent systematic review reported that brief interventions targeting long-term BZRA use in primary care (e.g., short consultations, written letters to patients) were effective in helping patients to discontinue BZRA medication. However, the complexity of these interventions has not been examined in detail. This study aimed to apply the intervention Complexity Assessment Tool for Systematic Reviews (iCAT_SR) to brief interventions targeting long-term BZRA use.

**Methods:**

Two reviewers independently assessed the interventions using the six core iCAT_SR dimensions: organisational level/ category targeted, behaviour targeted, number of intervention components, degree of tailoring, skill level required by those delivering and receiving the intervention. The four optional iCAT_SR dimensions were applied where possible. A scoring system was using to calculate a complexity score for each intervention. Pearson’s correlations were used to assess the relationship between intervention complexity and effect size, as well as the relationship between intervention complexity and number of component behaviour change techniques (BCTs). Inter-rater reliability was calculated using Cohen’s Kappa coefficient.

**Results:**

Four of the six core iCAT_SR dimensions were applied to the interventions with high inter-rater reliability (Cohen’s Kappa = 0.916). Application of the four optional dimensions was prevented by a lack of detail in study reports. Intervention complexity scores ranged from 8 to 11 (median: 11). There was no relationship detected between intervention complexity and either intervention effect size or number of component BCTs.

**Conclusions:**

This study adds to the literature on worked examples of the practical application of the iCAT_SR. The findings highlight how more detailed reporting of interventions is needed in order to optimise the application of iCAT_SR and its potential to differentiate between interventions across the full range of complexity dimensions. Further work is needed to establish the validity of applying a scoring system to iCAT_SR assessments.

**Supplementary Information:**

The online version contains supplementary material available at 10.1186/s12875-022-01775-y.

## Background

Benzodiazepine receptor agonists (BZRAs) are a class of drugs which include both benzodiazepines and Z-drugs (e.g. zopiclone, zolpidem). Benzodiazepines are indicated for numerous conditions, including anxiety and insomnia, whereas Z-drugs are solely indicated for the treatment of insomnia [1]. Clinical guidelines recommend that BZRA prescriptions should be restricted to short-term use (2–4 weeks), in order to reduce the risk of adverse effects [1–3]. Tolerance can occur over longer periods of use, whereby the patient experiences reduced effects from the BZRA medication with continued use [4]. Dependence can also develop after only a few weeks of regular BZRA use, and can result in withdrawal symptoms if patients attempt to discontinue the medication [3, 5]. Furthermore, long-term BZRA use (> 4 weeks) has been associated with a number of adverse outcomes such as cognitive impairment, unwanted sedation, and psychomotor impairment which, in turn, can lead to falls and fractures [6–8]. Despite these risks, BZRAs are still frequently prescribed on a long-term basis with many countries reporting either no changes or only small decreases in BZRA prescribing in recent years [9–11]. Older patients (≥ 65 years), who account for a substantial proportion of BZRA prescriptions, are particularly vulnerable to BZRAs’ adverse effects due to age-related changes in pharmacokinetic and pharmacodynamic processes, such as reduced drug metabolism and elimination, and increased sensitivity to BZRAs’ pharmacological effects [8, 12–14].

A number of strategies have been used to try to reduce long-term BZRA use [15–17], including brief interventions [18]. These generally refer to time-limited efforts to deliver advice or information to patients with the objective of eliciting change in their behaviour [19]. A recent systematic review by Lynch et al. [18] examined the effect of brief interventions targeting long-term BZRA use in primary care. The interventions evaluated in this review included written letters issued to patients recommending that they reduce their use of BZRAs, self-help booklets, and short consultations with healthcare professionals such as general practitioners and pharmacists providing information regarding BZRA dosage reduction. The review found that patients who received a brief intervention in primary care were more likely to reduce or discontinue long-term BZRA use after six to 12 months, compared to those who received usual care. This review also examined the intervention’s components using the Behaviour Change Technique Taxonomy (BCTTv1). Behaviour change techniques (BCTs) refer to the active components of an intervention which are designed to change behaviour [20]. BCTTv1 comprises 93 BCTs and can be applied to systematic reviews in order to identify interventions’ active components and explore their impact on intervention effectiveness. Seventeen BCTs were identified across the included studies with the number of BCTs per intervention ranging between 4–8 [18]. The most commonly identified BCTs in these interventions were ‘information about health consequences’, ‘credible source’ and ‘adding objects to the environment’. The review found no correlation between the number of identified BCTs and intervention effect size [18].

There is an increasing focus in health services research on complex interventions with several definitions discussed in the literature [21–26]. Complex interventions are frequently described as having non-linear causal pathways; this is in contrast with simple interventions which are commonly seen as having a linear pathway which links the intervention and the outcome [21, 26]. Other key aspects of complex interventions that have been identified are that they are context dependent and their components are interdependent [27]. The UK Medical Research Council (MRC) published a framework for developing, evaluating and implementing complex interventions which has been highly influential in this area [22, 27]. The MRC framework defines complex interventions as interventions containing several interacting components and outlines various dimensions of complexity. These include: the number of interacting components, the difficulty of behaviours needed by those receiving and delivering the intervention, the number of organisational levels targeted, variability of outcomes, and the degree to which flexibility or tailoring of the intervention is required [22]. Clarke et al. suggest that an intervention is complex when many factors are interacting dynamically, making it difficult to predict future outcomes based on past performance [25]. Some authors have suggested that no intervention can truly be categorised as ‘simple’ or ‘complex’ and that even seemingly simple interventions are often found to have complex aspects when they are examined closely [21].

The intervention Complexity Assessment Tool for Systematic Reviews (iCAT_SR) was developed by to provide systematic reviewers with a structured method to assess the complexity of an intervention [27]. The tool allows reviewers to identify and characterise key aspects of intervention complexity. The tool is made up of six core dimensions and four optional dimensions (Table 1). The six core dimensions include: number of active components, number of behaviours targeted, targeted organisational levels, degree of tailoring or flexibility of the intervention permitted, skills required by those delivering the intervention, and skills required by those receiving the intervention. The four optional dimensions are: degree of interaction between intervention components, the degree to which interventions are context dependent, the degree to which recipient or provider factors impact the intervention, and the nature of the causal pathway between intervention and outcome. Each dimension can be graded on one of three levels of increasing complexity [27]. An accompanying guidance document has also been developed which provides an in-depth explanation of each of the tool dimensions to assist reviewers in applying the tool [28].

**Table 1:** iCAT_SR dimensions and assessment categories [27, 29]

**Core Dimensions**	**Assessment Categories**
1. Organisational levels and categories targeted by the intervention	I. Single category II. Multi-category III. Multi-level
2. Behaviour or actions of intervention recipients or participants to which the intervention is directed	I. Single target II. Duel target III. Multi target
3. Active components included in the intervention, in relation to the comparison	I. One component II. More than one component III. More than one component *and* delivered a bundle
4. The degree of tailoring intended or flexibility permitted across sites or individuals in applying or implementing the intervention	I. Inflexible II. Moderately tailored/ flexible III. Highly tailored/ flexible
5. The level of skill required by those delivering the intervention in order to meet the intervention’s objectives	I. Basic skills II. Intermediate level skills III. High level skills
6. The level of skill required for the targeted behaviour when entering the included studies by those receiving the intervention, in order to meet the intervention’s objectives	I. Basic skills II. Intermediate level skills III. High level skills
**Optional Dimensions**	**Assessment Categories**
7. The degree of interaction between intervention components, including the independence / interdependence of intervention components	I. Independent II. Moderate interaction III. High level interaction
8. The degree to which the effects of the intervention are dependent on the context or setting in which it is implemented	I. Independent II. Moderately context dependent III. Highly context dependent
9. The degree to which the effects of the intervention are modified by recipient or provider factors	I. Largely independent of individual-level factors II. Moderately dependent on individual-level factors III. Highly dependent on individual-level factors
10. The nature of the causal pathway between the intervention and the outcome it is intended to effect	I. Pathway linear, short II. Pathway linear, long III. Pathway variable, long

A small number of worked examples involving application of the iCAT_SR to studies included in systematic reviews have been published to date [29–32]. For example, members of the research team previously applied the iCAT_SR to interventions aimed at improving appropriate polypharmacy in older people [29]. However, additional worked examples are needed to further enhance and refine the tool, as well as our understanding of how to optimise its application.

As brief interventions targeting long-term BZRA use provide an effective strategy for addressing a long-standing clinical issue, it is important to gain a more detailed understanding of these interventions and how they work. Many of the interventions examined in the previous systematic review were composed of several different components, making them “complex” [18]. However, the complexity of these interventions has not yet been comprehensively evaluated. The aim of this study was to apply the iCAT_SR [27] to studies included in a systematic review of brief interventions targeting long-term BZRA use in primary care [18]. The study objectives were to:

- Examine the complexity of the interventions using the iCAT_SR;
- Examine correlation between intervention complexity and intervention effect size;
- Examine the relationship between intervention complexity and number of BCTs identified in the interventions.

## Methods

We used the iCAT_SR [27] to assess the complexity of 12 interventions across eight studies [33–40] included in a recent systematic review of brief interventions targeting long-term BZRA use [18]. The interventions included in this review were targeted at changing patient’s long-term BZRA use behaviour and were carried out in primary care settings. The patients involved in these studies were 18 years and older (no upper age limit) and had been prescribed BZRAs on a long-term basis (≥ 3 months). For the four studies which assessed more than one brief intervention [34–36, 40], we applied the iCAT_SR dimensions to each intervention separately.

Two reviewers (AB, CC) in our team carried out the intervention complexity assessments independently using the iCAT_SR. For each study, we extracted the key information from the study using a data extraction form (Additional File 1). This form used was designed and applied in a previous related study by Cadogan et al. [29]. We used the iCAT_SR guidance document as a coding manual while carrying out the assessments [28]. This document outlines the criteria for each assessment level and provides relevant examples. We identified the relevant information for each assessment dimension within each of the study reports, assessed the complexity and provided support for our judgements to enhance transparency. Where there were differences in assessments between the two reviewers, we discussed these until consensus was reached. We applied the six core iCAT_SR dimensions to make assessments of each intervention. We also applied the four optional dimensions where possible.

We planned to base our assessments of skill level requirements on the reported details of prior training. However, the eight included studies did not explicitly report information related to the skills-related dimensions of the iCAT_SR (dimensions 5 and 6). Specifically, formal assessments of skill levels relating to BZRA discontinuation by either intervention deliverer or recipient were not documented in the study reports. To ensure consistency in our interpretation and assessment of these skills-related dimensions in the absence of explicit information, we therefore made a priori decisions on how to grade these dimensions. Our decisions were informed by our previous work involving application of the iCAT_SR [29]. We agreed that dimension 5 (level of skill required by the person delivering the intervention) would be classified as “intermediate skill level” for all of the interventions delivered by healthcare professionals, as supporting patients in successfully withdrawing from and discontinuing BZRAs is a challenging task. However, most of the required skills are likely already within the scope of practice of primary care-based healthcare professionals such as general practitioners and pharmacists and thus only modest upskilling would be required in order to meet the intervention objective. We also decided that dimension 6 (level of skill required by those receiving the intervention) would be classified as intermediate level skill across all of the interventions. This, in our judgement, reflects the challenges associated with successfully discontinuing BZRAs following prolonged use. Withdrawal symptoms associated with BZRA discontinuation have been widely reported and it has been estimated that between 15 and 44% of those who take benzodiazepines on a long-term basis will experience these symptoms [41]. Gradual dosage reduction was recommended in all of the interventions in included studies, and while this method may decrease withdrawal symptom severity, it does not prevent their occurrence [42]. We therefore assumed that an intermediate level of skill would have been required from patients in order for them to successfully discontinue BZRA use.

We used a colour scheme to allow visual representation of the different complexity dimensions assessed for each intervention (red = high level complexity, yellow = intermediate level complexity, green = low level complexity). We also applied a scoring system to numerically represent the complexity of the interventions. The scoring system that we used was employed in two recent systematic reviews [30, 31]. In this system, the intervention complexity score was calculated by the sum of the individual rating scores as follows: high level complexity = 3, intermediate level complexity = 2, low level complexity = 1. We only applied this scoring system to the six core dimensions due to challenges in assessing the four optional dimensions (discussed further in the results section below). Each intervention was therefore given a total score out of 18. We used Cohen’s Kappa co-efficient of inter-rater reliability to assess the level of agreement between the two reviewers based on their final individual assessments prior to consensus discussion (< 0 = poor agreement, 0—0.20 = slight agreement 0.21—0.40 = fair agreement, 0.41—0.60 = moderate agreement, 0.61—0.80 = substantial agreement, 0.81—1.00 = almost perfect agreement) [43].

In order to assess the potential utility of this scoring system, we undertook two exploratory analyses using the intervention complexity scores that we calculated. Firstly, to explore the relationship between intervention complexity and intervention effect size, we used Pearson’s correlation (two-tailed). The risk ratio for BZRA discontinuation at six months post-intervention was used as the measure of each interventions’ effect size as this outcome was reported across all eight included studies. These risk ratios were taken from those reported in the original systematic review for the included primary studies [18]. Secondly, we used Pearson’s correlations to explore the relationship between intervention complexity and the number of BCTs identified by Lynch et al. [18]. We conducted all statistical analyses using SPSS Version 27 (SPSS Inc. Chicago, IL, USA).

## Results

### Summary of included studies

An overview of each of the included studies and interventions to which the iCAT_SR was applied is provided in Table 2. Six of these studies were randomised controlled trials and two were cluster randomised controlled trials. The included studies comprised four two-armed trials and four three-armed trials. A total of 2,071 patients were involved across the eight studies. The majority of participants were female (71.2%) and participants’ mean age ranged from 59 to 75 years. The studies were carried out across four countries: Canada, Spain, United Kingdom, and United States.

**Table 2:** Overview of included studies and interventions

Study ID	Design	Participants and setting	Description of interventions and control
Bashir 1994 [33]	Two-armed randomised controlled trial	109 patients were recruited from eleven general practices 61.5% female Mean age = 62 years	Intervention group (*n* = 51): Patients attended consultation with their GP during which they were informed about the risks of benzodiazepines and advised to reduce benzodiazepine intake. The patients were also given a self-help booklet to take home which consisted of basic information about benzodiazepines and practical advice about stopping including techniques to manage fear and anxiety Control (*n* = 58): No intervention received
Cormack 1994 [34]	Three-armed randomised controlled trial	209 patients recruited from three general practices 79.4% female Mean age = 69 years	Intervention group 1 (*n* = 65): Letter signed by patients GP was sent to patients asking that they reduce or stop benzodiazepine use and advising to do so gradually Intervention group 2 (*n* = 75): Patients were sent same letter as group 1 as well as four information sheets at monthly intervals which provided practical advice about reducing benzodiazepines and managing without benzodiazepines Control (*n* = 69): No intervention received
Heather 2004 [35]	Three-armed randomised controlled trial	284 patients recruited from seven general practices 74% female Mean age = 69 years (standard deviation = 11.5)	Intervention Group 1 (*n* = 98): Letter was sent to patients in which they were invited to visit their general practitioner for a medication review. Guidelines were made which provided information for patient about benzodiazepines and the potential benefits of reducing their medication and a timetable to help the patients to plan their dose reduction. Patients also received a self-help booklet and an information sheet about sleeping problems Intervention Group 2 (*n* = 93): Patients received same letter from their general practitioner advising them to reduce or stop using benzodiazepines and that they should do so gradually. These patients did not receive the self-help booklet or the information leaflet Control (*n* = 69): No intervention received
Kuntz 2019 [36]	Three-armed randomised controlled trial	149 patients who were part of an integrated healthcare delivery system were recruited 66.4% female Mean age = 70 years	Intervention group 1 (*n* = 50): Letter sent to patients from prescriber in which they were advised to reconsider their Z-drug use. They were also provided with an educational brochure which gave information about the dangers associated with Z-drug use as well as proposals for other treatment options (pharmacological and non-pharmacological). They also received a tapering plan and a self-assessment quiz which reiterated the information from the brochure Intervention group 2 (*n* = 49): Same intervention as group 1 was given. These patients also received a follow up phone call from a clinical pharmacist two to four weeks after. The pharmacist reinforced the advice from the brochure and discussed obstacles to Z-drug discontinuation. They also provided advice on tapering off Z-drugs and made recommendations about alternatives to Z-drugs (such as sleep medicine). The pharmacist was given prescriber approval to implement a protocol to switch patients to safer medicines for insomnia treatment Control (*n* = 50): Standard care was provided
Navy 2018 [37]	Two-armed randomised controlled trial	364 patients who were members of an integrated healthcare delivery system were recruited 64% female Mean age = 73 years	Intervention group (n = 173): Letter was sent to patients from clinical pharmacist which advised about the risks associated with long-term use of alprazolam. The letter advised patients to call the clinical pharmacist to discuss reducing their alprazolam intake and other potential treatment options. Patients were told to not stop taking alprazolam without first consulting with the clinical pharmacist. The pharmacist worked collaboratively with the patient’s physician to develop an individualised dose reduction plan. The patients progress was monitored by the pharmacist through follow-up telephone calls Control (*n* = 173): Usual care was provided
Tannenbaum 2014 [38]	Two-armed cluster randomised controlled trial	303 patients recruited from 30 community pharmacies which were part of a chain 69% female Mean age = 75 years (standard deviation 6.3)	Intervention group (*n* = 148): Patients were sent a personalised educational leaflet. The booklet included a self-assessment about the risks associated with benzodiazepine use, information regarding benzodiazepine-induced harms, knowledge statements which were designed to create cognitive dissonance about the safety of benzodiazepine use, information about drug interactions, peer champion stories which aimed to enhance self-efficacy, proposals for therapeutic substitutes for insomnia and anxiety, and step-by-step tapering recommendations with illustrations. The intervention also asked participants to talk to their physician or pharmacist about these benzodiazepine deprescribing recommendations Control (*n* = 155): Received standard care. Educational intervention was provided 6 months later
Vicens 2006 [39]	Two-armed randomised controlled trial	139 patients recruited from three public primary care centres 82% female Mean age = 59 years (standard deviation = 11.4)	Intervention group (*n* = 73): GPs provided consultation to patients which consisted of a standardised message on the long-term risks of benzodiazepines and information on discontinuing benzodiazepines. Patients underwent gradual reduction of benzodiazepine use with fortnightly dosage reductions of 10–25% Control (*n* = 66): Usual care provided and the patients were advised about the convenience of reducing their benzodiazepine use
Vicens 2014 [40]	Three-armed cluster randomised control trial	532 patients recruited from 21 primary care centres 72% female Median age = 64 (range 55–72)	Intervention group 1 (*n* = 191): Patients received consultation from their GP and were provided with advice about risks of long-term BZRA use and reassured about reducing their use of their medication. Patients with insomnia were provided with a self-help leaflet regarding improving sleep quality. Patients gradually reduced their dose by 10–25% every 2–3 week at follow up consultations. GPs were allowed to switch patients who were experiencing withdrawal symptoms to long acting benzodiazepines Intervention group 2 (*n* = 168): Same initial GP consultation provided to patients as in group 1. This was followed by written instructions which reinforced the educational information and included an individually tailored gradual dose reduction plan. There were no follow up interviews scheduled but patients were permitted to request an appointment with their general practitioner if needed Control (*n* = 173): Patients received standard care

### Intervention complexity assessments

An overview of intervention complexity assessments for the six core iCAT_SR dimensions and the associated scores for each intervention are summarised in Table 3 and below. The full assessments for each intervention are provided in Additional file 2. Attempts were also made to apply the four optional iCAT_SR dimensions. However, it was not possible to apply each of these dimensions to the interventions due to a lack of detail in the study reports. Two studies reported on the impact of patient factors on intervention effectiveness [38, 40], allowing us to assess dimension 9. However, we were unable to assess dimensions 7, 8 or 10 for any of the studies.

**Table 3:**
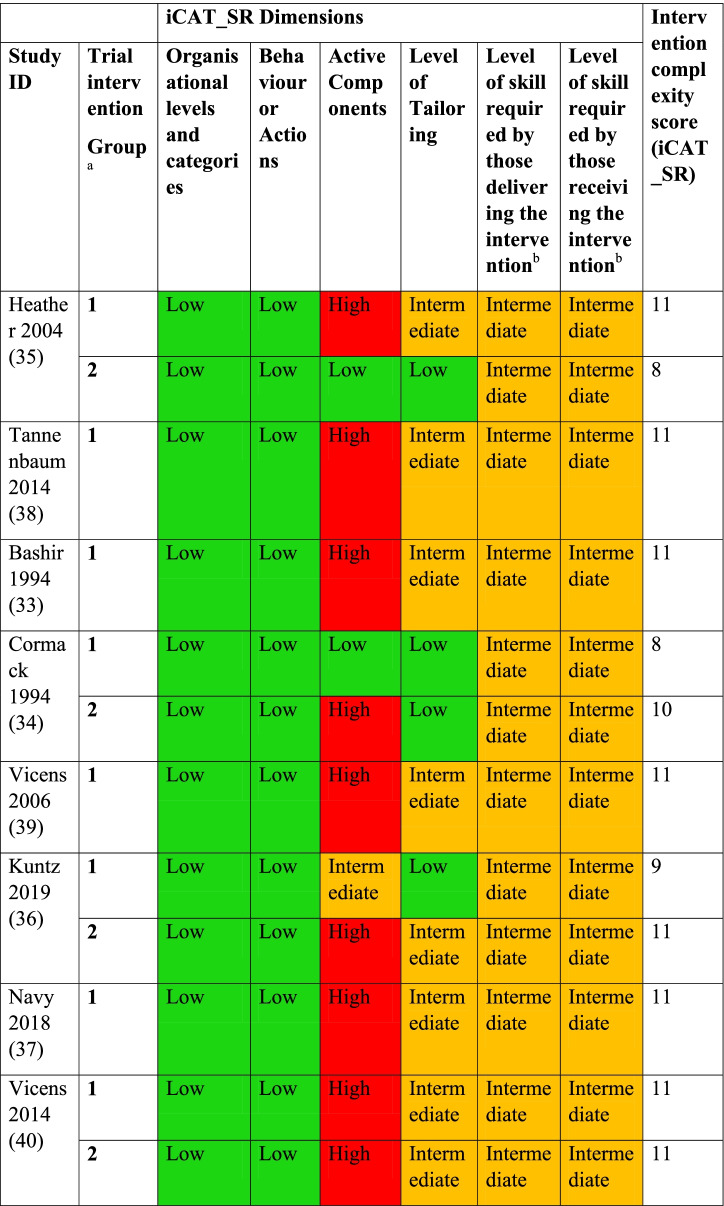
Assessments of core iCAT_SR dimensions and complexity score for each intervention

^a^Four included studies involved > 1 intervention group;

^b^Complexity rating agreed a priori

Inter-rater reliability was almost perfect (Cohen’s kappa = 0.916), indicating a very high level of agreement between the two reviewers across four of the six core dimensions (i.e., excluding dimensions 5 and 6 for which a priori decisions were made) based on their final individual assessments prior to consensus discussion.

#### Target organisation levels/categories and target behaviour/actions

All of the interventions targeted patients taking BZRAs on a long-term basis (single level category, low level complexity rating). All of the interventions were also considered to target a single behaviour/action (i.e., discontinuation of long-term BZRA use, low level complexity rating).

#### Active components

Nine interventions [33–40] were comprised of more than one component delivered as a bundle (high level complexity rating) due to the fact that the components were carried out in a series of steps. For example, some of these interventions consisted of consultations with a healthcare professional supplemented by a self-help booklet and tapering recommendations. For these interventions, a specific order in which components were to be delivered was implied in the study reports.

Only one intervention [36] was considered to be made up of more than one component and delivered as an integrated package (intermediate level complexity rating). For this intervention, the patients were sent a package consisting of a letter and an information brochure containing a self-assessment quiz. There was no specific order or timeframe in which the components had to be delivered evident in the study report.

Two of the interventions [34, 35] consisted of just one component (low level complexity rating). Both of these interventions involved a written letter from patients’ general practitioner which recommended that they reduce/discontinue their BZRA use.

#### Level of flexibility or tailoring

Eight interventions [33, 35–40] were classified as moderately tailored or flexible (intermediate level complexity rating). A number of these interventions involved consultations with a healthcare professional who was allowed some degree of flexibility in terms of the consultation format, while other interventions involved BZRA tapering plans which were specially tailored to the individual patient’s needs.

Four interventions [34–36] were categorised as being inflexible (low level complexity rating) in that the intervention was highly standardised with limited variation across participants. These interventions usually involved a standardised letter or information sheet being sent out to all participants with no variation in the material received by patients.

#### Level of skill required by those delivering and receiving interventions

Across all studies, the individuals who were responsible for delivering the intervention were deemed to require intermediate level skills (intermediate level complexity rating). The level of skill required for the targeted behaviour by those receiving the intervention was classed as being intermediate (intermediate level complexity rating).

#### Intervention complexity scores

The complexity scores calculated for each intervention are provided in Table 3. The median complexity score was 11 (range 8–11). The two interventions with the lowest complexity score of 8 involved a letter from the patient’s general practitioner [35] and a letter and information sheets being sent to patients [34]. The remaining ten interventions involved multiple intervention components, and allowed a moderate degree of flexibility in how they were carried out and therefore had higher complexity scores [33, 35–40].

### Relationship between intervention complexity and intervention effect size

As shown in Table 4, there was no detectable correlation between intervention complexity and effect size across 12 interventions.

**Table 4:** Discontinuation of BZRA use at 6 months post-intervention: Relationship between intervention effect size and intervention complexity score

**Study ID**	**Trial intervention Group**^a^	**Intervention Complexity Score (iCAT_SR)**	**Risk Ratio (as reported in the systematic review)**
**Bashir 1994** [33]	1	11	3.30
**Cormack 1994 **[34]	1	8	4.07
	2	10	5.67
**Heather 2004 **[35]	1	11	1.61
	2	8	1.575
**Kuntz 2019 **[36]	1	9	2.15
	2	11	2.12
**Navy 2018 **[37]	1	11	1.36
**Tannenbaum 2014 **[38]	1	11	5.52
**Vicens 2006 **[39]	1	11	13.11
**Vicens 2014 **[40]	1	11	2.97
	2	11	2.58
**Pearson correlation value**		0.175 (*p* = 0.587)	

^a^Four included studies involved > 1 intervention group

### Relationship between number of BCTs and intervention complexity

The number of BCTs identified for each intervention is outlined in Table 5 [18]. There was no detectable correlation between intervention complexity and the number of identified BCTs across 12 interventions.

**Table 5:** Relationship between intervention complexity and number of identified behaviour change techniques

**Study ID**	**Intervention Group**^a^	**Intervention Complexity Score (iCAT_SR)**	**Number of behaviour change techniques**
**Bashir 1994 **[33]	1	11	5
**Cormack 1994 **[34]	1	8	7
	2	10	8
**Heather 2004 **[35]	1	11	5
	2	8	6
**Kuntz 2019 **[36]	1	9	4
	2	11	6
**Navy 2018 **[37]	1	11	6
**Tannenbaum 2014 **[38]	1	11	6
**Vicens 2006 **[39]	1	11	5
**Vicens 2014 **[40]	1	11	7
	2	11	6
**Pearson correlation value**		-0.121 (*p* = 0.708)	

^a^Four included studies involved > 1 intervention group

## Discussion

In this study, we used the iCAT_SR to assess the complexity of brief interventions targeting long-term BZRA use in primary care across eight studies [33–40]. This builds on the findings from the existing systematic review by Lynch et al. [18] which identified component BCTs but did not examine the complexity of the interventions any further. This study adds to the literature on worked examples of the practical application of the iCAT_SR [29]. Our previous work involved the tool’s application to interventions aimed at improving appropriate polypharmacy in older people [29] which had different targets and were conducted in different clinical settings. In contrast, the interventions that were examined in the current study targeted patients taking BZRAs on a long-term basis in primary care settings and a single behaviour/action (i.e., BZRA discontinuation). This enabled application of iCAT_SR to a more homogenous group of interventions.

This homogeneity across interventions was evident in the assessments relating to two of the tool’s core domains: ‘Organisational levels and categories’ (i.e., patients taking BZRAs on a long-term basis in primary care settings) and ‘Behaviour or Actions’ (i.e., BZRA discontinuation). As noted earlier, we made a priori decisions in relation to the scoring of these two core domains involving skill level requirements. For instance, patients receiving the interventions were deemed to require “intermediate level skills” due to the difficulty associated with discontinuing long-term BZRA use and the possibility of withdrawal symptoms [41, 42]. We view this as an aspect of complexity that arises in all brief interventions with the same primary outcome of reducing or discontinuing long-term BZRA use. While our assessments for these two domains may have limited the full potential to critically discriminate between interventions using iCAT_SR, we believe that our choices are justifiable given the level of reporting in the included studies.

With regard to the domains focusing on active components and level of tailoring, our study highlights some important similarities and differences between the interventions across the included studies. We found that almost all of the interventions that had multiple active components allowed for some degree of tailoring. In most cases these interventions involved a consultation between the patient and healthcare professional where the information and advice could be tailored according to individual patient needs. The only exception was the study by Cormack et al. [34] which involved a three-armed trial whereby patients assigned to either one of two interventions groups were compared against usual care. In the first group, patients received a letter from their GP asking them to consider discontinuing BRZRA use and advising that this should be done gradually. In the second group, patients received the same letter as the first intervention group, followed at monthly intervals by written information sheets giving advice about BZRA dosage reduction. Although the second intervention had multiple components, it was considered to involve a low level of tailoring as all patients received the same information sheets. Flexible interventions are important for promoting BZRA discontinuation, with many studies and guidelines recommending that BZRA dose reduction should be tailored according to the needs and ability of individual patient to tolerate withdrawal symptoms [4, 44, 45].

For most of the studies, it was not possible to assess interventions in terms of the four optional dimensions due to the lack of detailed intervention reporting in the included primary studies – a limitation noted in the original systematic review [18]. This lack of detailed intervention reporting has also hampered efforts to apply these dimensions in previous research involving the iCAT_SR [29]. The need for a more robust approach to the development, evaluation, implementation and reporting of complex interventions, including a theoretical understanding of the causal pathway and how the intervention brings about change, is widely recognised [22, 46, 47]. Enhanced reporting of causal pathways between interventions and outcome would allow greater understanding of how the intervention is intended to work and whether these hypothesised pathways can be substantiated in practice. As many interventions targeting long-term BZRA use are composed of multiple components, it is also important to have an understanding of how intervention components interact with each other and whether or not this has an impact on effectiveness. More detailed reporting on the intervention recipients would aid in identifying patients who are similar to those who benefited from the interventions in the trials, and in establishing which types of intervention are most effective in relation to specific patient characteristics. Furthermore, identifying intervention deliverer factors that impact on intervention effectiveness would help in determining the most appropriate individuals to deliver the intervention. It is well understood that replication requires detailed reporting [24, 48], and consequently, improved reporting of the complexity dimensions for brief intervention targeting BZRA use would be a pivotal step in allowing future implementation and scale up across health systems. We acknowledge, though, that many other factors affect the implementation of health interventions, including the acceptability of these to those involved and their feasibility within the implementation context [22, 49, 50].

Previous work involving iCAT_SR has applied a scoring system to each assessment dimension and level of complexity to quantify intervention complexity. These scoring systems have been used to examine the relationship between intervention complexity and effectiveness [30–32, 51]. To attempt to provide novel insights into how the iCAT_SR assessments could be used in conjunction with effect estimates and methodological innovations in systematic reviews (e.g., BCT coding), we explored the use of the scoring system applied in previous research [30–32, 51]. No correlation was detected between intervention effectiveness and complexity across the twelve interventions assessed as part of this study. This is consistent with previous research involving application of iCAT_SR [30, 31]. Similar findings were observed when the relationship between the number of component BCTs and complexity assessment scores was examined. The lack of correlation that we observed may have been attributable to a number of factors. Firstly, the modest number of interventions and narrow range in complexity scores decreased the likelihood of a detectable relationship being found between intervention complexity and intervention effectiveness [52]. Secondly, because we were not able to implement the four optional dimensions, a full assessment of intervention complexity was not possible. This potentially limits interpretation of our findings regarding the relationship between complexity and intervention effect size and between number of component BCTs and complexity. Further methodological research exploring how the BCT taxonomy can aid future refinement of the iCAT_SR may be helpful.

Complexity scores did not vary substantially across the studies based on the current scoring system. This is perhaps unsurprising, as all of the studies were focused on brief interventions for the reduction/discontinuation of long-term BZRA use. The only complexity aspects that differed across the interventions were the number of intervention components and the degree of tailoring of the intervention. As noted above, the provision of information with respect to the four optional dimensions would have enhanced the potential to differentiate between the interventions in terms of complexity.

The validity and usefulness of applying using a scoring system as part of the application of iCAT_SR remains to be determined. Potential limitations of doing so are that such scoring may provide an overly simplistic view of intervention complexity. In addition, each complexity dimension carried equal weighting in the scoring system that was used, and it may be the case that some complexity questions should be given more weight than others. However, scoring the assessments can facilitate efforts to quantitatively explore relationships between intervention effect size and intervention complexity. This, in turn, may be helpful for understanding the extent to which effects are dependent on higher levels of intervention complexity. Further work is therefore needed to explore whether it is possible to validate a scoring system for the iCAT_SR. In the interim, we suggest that future applications of the iCAT_SR avoid an oversimplistic scoring system to reduce the risk of inaccurate conclusions being drawn being drawn regarding the relationship between intervention complexity and intervention effects.

### Strengths and limitations

This study provides an additional worked example of the application of iCAT_SR, contributing to the continuing development and refinement of the tool. There are limited examples of the tool’s application to date [29] and this study further highlights key issues that researchers need to be aware of before attempting to apply it as part of a systematic review. The main limitation of this study is the relatively small number of interventions available for assessment (*n* = 12). Additional limitations are the reliance on published study reports and, linked to this, inadequate reporting detail in these papers to implement the four optional iCAT_SR dimensions. Further, as the review on which this study is based included a relatively narrow range of interventions, it is possible that the tool is not sufficiently sensitive to identify variations in intervention complexity within this range.

Our study makes an important contribution to published experiences of applying the iCAT_SR tool, and to identifying areas where further methodological research is needed. iCAT_SR assessments are judgements and the guidance for use of the tool encourages researchers to provide support for their judgements to both help ensure transparency and help others understand these judgements. We have tried to do that for our assessments, but acknowledge that others may have reached different judgements, particularly in relation to the ICAT_SR dimensions 5 and 6 focusing on the level of skill required by those delivering the intervention in order to meet the intervention objective and the level of skill required for the targeted behaviour when entering the included studies by those receiving the intervention, in order to meet the intervention objectives. In particular, not all readers may agree with our decision to standardise the measurement scores for dimensions 5 and 6 and rank all interventions as “intermediate skill”, and our approach limited the range of variation possible in our complexity scores. Our decision was based in part on limited intervention reporting in the included studies, as noted elsewhere in this paper.

Another important limitation is the uncertainty regarding validity and usefulness of the current scoring system as a method for quantifying intervention complexity. In the current approach, the overall score is a continuous variable and may not account appropriately for the variance within and across domains. Also, the scoring system gives each domain equal weight for the purposes of aggregation and it is not yet clear whether this is an appropriate approach. As noted above, we therefore suggest that future applications of the iCAT_SR avoid the use of a scoring system until it has been ascertained through methodological research whether a suitable system can be developed and validated.

As this was a secondary analysis of studies included in a previous systematic review, this work is not intended as a definitive assessment of brief interventions targeting long-term BZRA use in primary care but rather a worked example of the application of iCAT_SR. Further work to assess the complexity of these brief interventions would also need to involve updating the systematic review to identify any new evaluations. Finally, we recognise that there are a number of other tools and checklists available to improve the reporting of different aspects of interventions included in systematic reviews (for example, Hoffman 2017 [53]; Montgomery 2013 [54]). While these do not focus explicitly on assessing the complexity of interventions, they may have value in identifying and understanding related concepts.

## Conclusions

This study provides a detailed overview of the application of the iCAT_SR to brief interventions targeting long-term BZRA use in primary care. An understanding of the aspects of complexity which arise in brief interventions targeting BZRA use may be beneficial for future researchers developing new interventions targeting BZRA use. The iCAT_SR dimensions could also help researchers in conceptualising these interventions and aid intervention description in trial reports.

The findings highlight that more detailed reporting of interventions is needed in order to optimise the application of iCAT_SR and its potential to differentiate between interventions across the full range of complexity dimensions. In addition, our understanding of the usefulness of iCAT_SR in explaining variation in effect estimates across interventions addressing a particular health issue could be advanced by applying the tool within reviews with wider eligibility criteria and therefore greater variation in intervention complexity. However, further work is needed to determine the validity and usefulness of applying a scoring system to iCAT_SR assessments.

## Supplementary Information

**Additional file 1.** Data extraction form.

**Additional file 2.** Intervention complexity assessments based on the iCAT_SR tool. 

## Data Availability

The datasets used and/or analysed during the current study are available from the corresponding author on reasonable request.

## Abbreviations

BCT: Behaviour change technique
BZRA: Benzodiazepine receptor agonist
iCAT_SR: Intervention Complexity Assessment Tool for Systematic Reviews
MRC: Medical Research Council
