# Supplementary material for: Applying the intervention Complexity Assessment Tool to brief interventions targeting long-term benzodiazepine receptor agonist use in primary care: Lessons learned
Source: BMC Prim Care. 2022 Jul 16;23:175. doi: 10.1186/s12875-022-01775-y (PMC9288038; doi:10.1186/s12875-022-01775-y)
Supplement: Supplementary file 1 — Additional file 1. Data extraction form. [file 12875_2022_1775_MOESM1_ESM.pdf]

Additional File 1: Data extraction form

Study ID:
Study title:

**Study and Intervention details**			
Study aims/objectives			
Study outcome(s)			
Details of intervention/s and the comparison (including usual care)			
Intervention aim/objectives			
Intervention deliverer			
Intervention target/recipient (which may include individuals, groups of individuals and other entities)			
Target behaviour and component actions (i.e. whose behaviour the intervention intended to change and what were the component actions involved in that behaviour)			
**Core dimension**	**Description of the intervention in the review**	**Judgement**	**Support for judgement**
**1. Organisational levels and categories targeted by the intervention**		– Multi-level<br>– Multi-category<br>– Single category	
**2. Behaviour or actions of intervention recipients or participants to which the intervention is directed**		– Multi-target<br>– Dual target<br>– Single target<br>– Varies	
**3. Active components included in the intervention, in relation to the comparison**		– More than one component and delivered as a bundle<br>– More than one component<br>One component	
**4. The degree of tailoring intended or flexibility permitted across sites or individuals in applying or implementing the intervention**		– Highly tailored/flexible<br>– Moderately tailored/flexible	

		– Inflexible<br>– Varies	
**5. The level of skill required by those delivering the intervention in order to meet the intervention's objectives**		– High level skills<br>– Intermediate level skills<br>– Basic skills<br>– Varies	
**6. The level of skill required for the targeted behaviour when entering the included studies by those receiving the intervention, in order to meet the intervention's objectives**		– High level skills<br>– Intermediate level skills<br>– Basic skills<br>– Varies	
**Optional dimension**	**Description of the intervention in the review**	**Judgement**	**Support for judgement**
**7. The degree of interaction between intervention components, including the independence / interdependence of intervention components**		– High level interaction<br>– Moderate interaction<br>– Independent<br>– Varies<br>– Unclear/Unable to assess	
**8. The degree to which the effects of the intervention are dependent on the context or setting in which it is implemented**		– Highly context dependent<br>– Moderately context dependent<br>– Independent of context<br>– Varies<br>– Unclear/Unable to assess	
**9. The degree to which the effects of the intervention are modified by recipient or provider factors**		– Highly dependent on individual-level factors<br>– Moderately dependent on individual-level	

		factors<br>– Largely independent of individual-level factors<br>– Varies<br>– Unclear/Unable to assess	
**10. The nature of the causal pathway between the intervention and the outcome it is intended to effect**		– Pathway variable, long<br>– Pathway linear, long<br>– Pathway linear, short<br>– Varies<br>– Unclear/Unable to assess	
