# Supplementary material for: Applying the intervention Complexity Assessment Tool to brief interventions targeting long-term benzodiazepine receptor agonist use in primary care: Lessons learned
Source: BMC Prim Care. 2022 Jul 16;23:175. doi: 10.1186/s12875-022-01775-y (PMC9288038; doi:10.1186/s12875-022-01775-y)
Supplement: Supplementary file 2 — Additional file 2. Intervention complexity assessments based on the iCAT_SR tool. [file 12875_2022_1775_MOESM2_ESM.pdf]

Additional file 2: Intervention complexity assessments based on the iCAT_SR tool

Study ID: Heather 2004
Study title: Randomised controlled trial of two brief interventions against long-term benzodiazepine use: outcome of intervention

**Study and Intervention details**	
Study aims/objectives	"(i) to replicate the earlier findings in a single RCT and determine whether brief interventions were effective in a socio-economically deprived geographical area (Newcastle and North Tyneside); (ii) to compare directly the effectiveness of the letter and consultation interventions"
Study outcome(s)	"The main outcome measure was change in BENZODIAZEPINE intake between the six-month periods before and after the intervention"
Details of intervention/s and the comparison (including usual care)	**Consultation group**:"98 patients were sent a letter inviting them to see their GP for a medication review. Before the trial began, the researcher met participating GPs to give guidance on how the consultation should be carried out. Consultations were scheduled to last for 12 min. Written guidelines were produced consisting of information for patients about benzodiazepines, reasons why it might be beneficial to reduce medication and a timetable that could be used to plan withdrawal (see Appendix 1). These guidelines were attached to patients' notes so that the GP could refer to them during the consultation. GPs were allowed discretion as to how the consultation was conducted. Copies of a self-help booklet, entitled Helping you Cope: A Guide to Starting and Stopping Tranquillisers and Sleeping Tablets, were supplied by The Mental Health Foundation and given to patients during the consultation, along with a leaflet about sleeping problems. In one practice, the consultation was carried out by the Practice Pharmacist (27 patients) and in another by a Practice Nurse (3 patients)."<br><br>**Letter Group:** "In this group, 93 patients were sent an amended version of the letter used in the study by Cormack and colleagues (see Appendix 2). The letter was produced by the research team on practice-headed paper and signed by the patient's usual GP. Patients in the Letter group were not sent the self-help booklet or leaflet."<br><br>**Control group**: "The 93 patients in this group received usual care but no intervention"
Intervention aim/objectives	Benzodiazepine discontinuation
Intervention deliverer	**Consultation group:** GPs<br><br>**Letter Group:** Not reported

Intervention target/recipient (which may include individuals, groups of individuals and other entities)	"long-term BENZODIAZEPINE users who would be identified by their GPs as suitable to receive a brief intervention aimed at encouraging a reduction in BENZODIAZEPINE intake"		
Target behaviour and component actions (i.e. whose behaviour the intervention intended to change and what were the component actions involved in that behaviour	"change in BENZODIAZEPINE intake between the six-month periods before and after the intervention."		
**Intervention Group 1: Consultation Group**			
**Core dimension**	**Description of the intervention in the review**	**Judgement**	**Support for judgement**
**1. Organisational levels and categories targeted by the intervention**	"…patients were sent a letter inviting them to see their GP for a medication review.."	Single category	Intervention only targets patients
**2. Behaviour or actions of intervention recipients or participants to which the intervention is . directed**	"The main outcome measure was change in BENZODIAZEPINE intake between the six-month periods before and after the intervention."	Single target	Only one behaviour the intervention is targeting – benzodiazepine intake.
**3. Active components included in the intervention, in relation to the comparison**	"In this group, 98 patients were sent a letter inviting them to see their GP for a medication review. Before the trial began, the researcher met participating GPs to give guidance on how the consultation should be carried out. Consultations were scheduled to last for 12 min. Written guidelines were produced consisting of information for patients about benzodiazepines, reasons why it might be beneficial to reduce medication and a timetable that could be used to plan withdrawal (see Appendix 1). These guidelines were attached to patients' notes so that the GP could refer to them during the consultation. GPs were allowed discretion as to how the consultation was conducted. Copies of a self-help booklet, entitled Helping you Cope: A Guide	More than one component and delivered as a bundle	The components of the intervention included a GP consultation, self-help booklet, a leaflet, and a letter. Defined order in the delivery of these interventions – academic review would precede use of the web-based algorithm, medication review and distribution of PILs to patients.

	to Starting and Stopping Tranquillisers and Sleeping Tablets, were supplied by The Mental Health Foundation and given to patients during the consultation, along with a leaflet about sleeping problems. In one practice, the consultation was carried out by the Practice Pharmacist (27 patients) and in another by a Practice Nurse (3 patients).”		
**4. . The degree of tailoring intended or flexibility permitted across sites or individuals in applying or implementing the intervention**	“GPs were allowed discretion as to how the consultation was conducted.”	Moderately flexible	GPs were allowed discretion and could tailor consultations.
**5. The level of skill required by those delivering the intervention in order to meet the intervention’s objectives**	Before the trial began, the researcher met participating GPs to give guidance on how the consultation should be carried out. Consultations were scheduled to last for 12 min. Written guidelines were produced consisting of information for patients about benzodiazepines, reasons why it might be beneficial to reduce medication and a timetable that could be used to plan withdrawal (see Appendix 1). These guidelines were attached to patients’ notes so that the GP could refer to them during the consultation	Intermediate level skills	GPs had to be upskilled in how to carry out the consultations and support BZRA discontinuation .
**6. The level of skill required for the targeted behaviour when entering the included studies by those receiving the intervention, in order to meet the intervention’s objectives**	Not explicitly stated in the report – high level skills required to undergo successful BZRA discontinuation	High level skills	High level skills required to undergo BZRA discontinuation .
**Optional dimension**	**Description of the intervention in the review**	**Judgement**	**Support for judgement**

**7. The degree of interaction between intervention components, including the independence / interdependence of intervention components**	Not outlined	unclear/ unable to assess	
**8. The degree to which the effects of the intervention are dependent on the context or setting in which it is implemented**	Not outlined	Unclear/Unable to assess	
**9. The degree to which the effects of the intervention are modified by recipient or provider factors**	Not outlined	Unclear/ Unable to assess	
**10. The nature of the causal pathway between the intervention and the outcome it is intended to effect**	Not outlined	Unclear/Unable to assess	

**Intervention Group 2: Letter Group**			
**Core dimension**	**Description of the intervention in the review**	**Judgement**	**Support for judgement**
**1) Organisational levels and categories targeted by the intervention**	"…patients were sent a letter inviting them to see their GP for a medication review.."	Single category	Intervention only targets patients
**2) Behaviour or actions of intervention recipients or participants to which the intervention is directed**	"The main outcome measure was change in benzodiazepine intake between the six-month periods before and after the intervention."	Single target	Only one behaviour the intervention is targeting - benzodiazepine intake.
**3) Active components included in the intervention, in relation to the comparison**	"93 patients were sent an amended version of the letter used in the study by Cormack and colleagues (see Appendix 2). The letter was produced by the research team on practice-headed paper and signed by the patient's usual GP. Patients in the Letter group were not sent the self-help booklet or leaflet"	One component	Letter was the only component of the intervention.
**4) The degree of tailoring intended or flexibility permitted across sites or individuals in applying or implementing the intervention**	"patients were sent an amended version of the letter used in the study by Cormack and colleagues"	Inflexible	No tailoring of letter.
**5) The level of skill required by those delivering the intervention in order to meet the intervention's objectives**	Not explicitly stated – intermediate level skills needed to promote discontinuation of BZRA	Intermediate level skills	Intermediate level skills to promote discontinuation of BZRA.

**6) The level of skill required for the targeted behaviour when entering the included studies by those receiving the intervention, in order to meet the intervention's objectives**	Not explicitly stated in the report – high level skills required to undergo successful BZRA discontinuation	High level skills	High level skills required to undergo BZRA discontinuation .
**Optional dimension**	**Description of the intervention in the review**	**Judgement**	**Support for judgement**
**7) The degree of interaction between intervention components, including the independence / interdependence of intervention components**	Not outlined	unclear/ unable to assess	
**8) The degree to which the effects of the intervention are dependent on the context or setting in which it is implemented**	Not outlined	Unclear/Unable to assess	
**9) The degree to which the effects of the intervention are modified by recipient or provider factors**	Not outlined	Unclear/ Unable to assess	
**10) The nature of the causal pathway between the intervention and the outcome it is intended to effect**	Not outlined	Unclear/Unable to assess	

Study ID: Tannenbaum 2014

Study title: Reduction of Inappropriate Benzodiazepine Prescriptions Among Older Adults Through Direct Patient Education The EMPOWER Cluster Randomized Trial

**Study and Intervention details**	
Study aims/objectives	"to test the effectiveness of direct patient education about drug harms on benzodiazepine therapy discontinuation among community-dwelling adults 65 years and older receiving long-term benzodiazepine therapy"
Study outcome(s)	"The primary outcome was complete cessation of benzodiazepine use in the 6 months following randomization." *Results for secondary outcomes were not included in the paper.*
Details of intervention/s and the comparison (including usual care)	**Intervention:** "The patient empowerment intervention consisted of an 8-page booklet based on social constructivist learning and self-efficacy theory, and its development and testing have been previously detailed. The intervention comprises a self-assessment component about the risks of benzodiazepine use, presentation of the evidence for benzodiazepine-induced harms, knowledge statements designed to create cognitive dissonance about the safety of benzodiazepine use, education about drug interactions, peer champion stories intended to augment self-efficacy,

	suggestions for equally or more effective therapeutic substitutes for insomnia and/or anxiety, and stepwise tapering recommendations. Tapering recommendations consist of a visual 21-week tapering protocol showing a picture-based diminishing schedule of full-pill, half-pill, and quarter-pill consumption. The visual schematic for the deprescribing protocol was proposed by consumers during the development and usability testing of the intervention to enable application to any benzodiazepine, regardless of dose. The intervention asks participants to discuss the deprescribing recommendations with their physician and/or pharmacist. The information is included in a letter-size paper handbook, with the language set at a sixth-grade reading level and written in 14-point font to facilitate accessibility to the material. **The intervention was personalized according to the participant's pharmacy profile to include the name of the specific benzodiazepine the participants was taking**. The intervention was mailed to the intervention group within 1 week of group allocation while the usual care (wait list) group received the educational tool 6 months following group allocation. A full version of the intervention is available in the appendix in the Supplement."<br><br>**Control:** "The intervention was mailed to the intervention group within 1 week of group allocation while the usual care (wait list) group received the educational tool 6 months following group allocation. "
Intervention aim/objectives	"The intervention asks participants to discuss the deprescribing recommendations with their physician and/or pharmacist"
Intervention deliverer	Research team<br><br>"A full list of pharmacies within 200km of the research centre was obtained through collaboration with the pharmacy chain's headquarters. This list was randomized, and pharmacies were systematically contacted by the research team to assess interest in participating."<br><br>"All clients meeting study criteria received a recruitment mailing followed by telephone call invitations from their pharmacists. Patients who expressed interest in participating in the study were directed to the study team and screened for eligibility via in-home interviews with a research assistant."<br><br>"The intervention asks participants to discuss the deprescribing recommendations with their physician and/or pharmacist.""
Intervention target/recipient (which may include individuals, groups of individuals and other entities)	"The sampling frame for individual participants was a list of all adults 65 years and older receiving long-term benzodiazepine therapy from each participating pharmacy, provided to pharmacists by the central database system of the pharmacy chain. "
Target behaviour and component actions (i.e. whose behaviour the intervention intended to	"benzodiazepine therapy discontinuation among community-dwelling adults 65 years and older receiving long-term benzodiazepine therapy."

change and what were the component actions involved in that behaviour)	"discuss the deprescribing recommendations with their physician and/or pharmacist"		
**Core dimension**	**Description of the intervention in the review**	**Judgement**	**Support for judgement**
**1. Organisational levels and categories targeted by the intervention**	"a direct-to-consumer educational intervention"<br><br>"The intervention was mailed to the intervention group"	Single category	Intervention was only targeted at patients.
**2. Behaviour or actions of intervention recipients or participants to which the intervention is directed**	"benzodiazepine therapy discontinuation among community-dwelling adults 65 years and older receiving long-term benzodiazepine therapy." "discuss the deprescribing recommendations with their physician and/or pharmacist"	Single target	Only one behaviour being targeted - benzodiazepine discontinuation.
**3. Active components included in the intervention, in relation to the comparison**	"The patient empowerment intervention consisted of an 8-page booklet based on social constructivist learning and self-efficacy theory, and its development and testing have been previously detailed. The intervention comprises a self-assessment component about the risks of benzodiazepine use, presentation of the evidence for benzodiazepine-induced harms, knowledge statements designed to create cognitive dissonance about the safety of benzodiazepine use, education about drug interactions, peer champion stories intended to augment self-efficacy, suggestions for equally or more effective therapeutic substitutes for insomnia and/or anxiety, and	More than one component and delivered as a bundle	Intervention was delivered as a bundle consisting of a number of components - booklet, self-assessment, tapering recommendations etc.

	stepwise tapering recommendations. Tapering recommendations consist of a visual 21-week tapering protocol showing a picture-based diminishing schedule of full-pill, half-pill, and quarter-pill consumption." "The intervention asks participants to discuss the deprescribing recommendations with their physician and/or pharmacist."		
**4. The degree of tailoring intended or flexibility permitted across sites or individuals in applying or implementing the intervention**	"The intervention was personalized according to the participant's pharmacy profile to include the name of the specific benzodiazepine the participants were taking"	Moderately flexible	Some personalisation of intervention depending on individuals pharmacy profile and the name of the benzodiazepine.
**5. The level of skill required by those delivering the intervention in order to meet the intervention's objectives**	Skills not explicitly reported – intermediate level skills needed to promote/ support successful BRZA discontinuation.	Intermediate level skills	Intermediate level skills needed to promote/ support successful BRZA discontinuation
**6. The level of skill required for the targeted behaviour when entering the included studies by those receiving the intervention, in order to meet the intervention's objectives**	Skills not explicitly reported "Tapering recommendations consist of a visual 21-week tapering protocol showing a picture-based diminishing schedule of full-pill, half-pill, and quarter-pill consumption. The visual schematic for the deprescribing protocol was proposed by consumers during the development and usability testing of the intervention to enable application to any benzodiazepine,	High level sills	High level skills required to undergo successful BZRA discontinuation.

	regardless of dose. The intervention asks participants to discuss the deprescribing recommendations with their physician and/or pharmacist."		
**Optional dimension**	**Description of the intervention in the review**	**Judgement**	**Support for judgement**
**7. The degree of interaction between intervention components, including the independence / interdependence of intervention components**	Not stated	Unclear/Unable to assess	
**8. The degree to which the effects of the intervention are dependent on the context or setting in which it is implemented**	Not Stated	Unclear/Unable to assess	
**9. The degree to which the effects of the intervention are modified by recipient or provider factors**	"The effect of the intervention was robust across age, indication, dose, and duration of benzodiazepine use."	Largely independent of individual-level factors	Effect of intervention was not impacted by patient characteristics.
**10. The nature of the causal pathway between the intervention and the outcome it is intended to effect**	Not stated	Unclear/Unable to assess	

Study ID: Bashir 1994

Study title: Controlled evaluation of brief intervention by general practitioners to reduce chronic use of benzodiazepines

**Study and Intervention details**			
Study aims/objectives	"to assess the effectiveness of minimal intervention delivered by general practitioners in helping chronic users of benzodiazepines to withdraw from their medication, and to determine the psychological sequelae on patients of such intervention"		
Study outcome(s)	Withdrawal from benzodiazepines<br>Change in "psychological distress experienced before and after intervention"		
Details of intervention/s and the comparison (including usual care)	"Patients were allocated by their doctor to receive either minimal intervention, consisting of general practitioner advice on coming off benzodiazepines plus a self-help booklet which patients took away to read, or to receive no intervention: this group acted as controls."<br>"It would have been impossible in a controlled trial to impose rigid guidelines on general practitioners concerning the management of benzodiazepine withdrawal. Instead it was suggested that doctors should outline the risks of benzodiazepines, advise patients to reduce and then stop their medication, and then encourage patients to follow the advice in the self-help booklet. The booklet was divided into two sections, the first giving some basic information about benzodiazepines and the second giving practical advice on stopping, including techniques on coping with fears and anxieties."		
Intervention aim/objectives	"help chronic users to withdraw from their benzodiazepines."		
Intervention deliverer	GP – "It would have been impossible in a controlled trial to impose rigid guidelines on general practitioners concerning the management of benzodiazepine withdrawal"		
Intervention target/recipient (which may include individuals, groups of individuals and other entities)	"General practitioners were asked to recruit all chronic benzodiazepine users"<br>"A chronic user was defined as someone who had been on benzodiazepines for at least a year and who took tablets at least three times weekly."		
Target behaviour and component actions (i.e. whose behaviour the intervention intended to change and what were the component actions involved in that behaviour)	Patients "to withdraw from their medication" (benzodiazepines)		
**Core dimension**	**Description of the intervention in the review**	**Judgement**	**Support for judgement**
**1. Organisational levels and categories targeted by the intervention**	"Patients were allocated by their doctor to receive either minimal intervention,…"	Single category	Only the patients are targeted by the intervention.
**2. Behaviour or actions of intervention recipients or participants to which the intervention is directed**	"help chronic users to withdraw from their benzodiazepines."	Single target	Only one behaviour targeted –

			benzodiazepine use.
**3. Active components included in the intervention, in relation to the comparison**	"Patients were allocated by their doctor to receive either minimal intervention, consisting of general practitioner advice on coming off benzodiazepines plus a self-help booklet which patients took away to read, or to receive no intervention: this group acted as controls."	More than one component as a bundle	Intervention included advice from doctor and self-help book to take away.
**4. The degree of tailoring intended or flexibility permitted across sites or individuals in applying or implementing the intervention**	"It would have been impossible in a controlled trial to impose rigid guidelines on general practitioners concerning the management of benzodiazepine withdrawal. Instead it was suggested that doctors should …."	Moderately tailored/flexible	No rigid guidelines on how GP should manage benzodiazepine withdrawal.
**5. The level of skill required by those delivering the intervention in order to meet the intervention's objectives**	No explicitly mentioned in report – intermediate level skills to support patients in benzodiazepine withdrawal.	Intermediate level skills	Intermediate level skills to support patients in benzodiazepine withdrawal.
**6. The level of skill required for the targeted behaviour when entering the included studies by those receiving the intervention, in order to meet the intervention's objectives**	Not explicitly stated in the report – high level skills required to undergo successful BZRA discontinuation	High level skills	High level skill to undergo benzodiazepine discontinuation successfully.
**Optional dimension**	**Description of the intervention in the review**	**Judgement**	**Support for judgement**
**7. The degree of interaction between intervention components, including the independence / interdependence of intervention components**	Not outlined	Unclear/Unable to assess	
**8. The degree to which the effects of the intervention are dependent on the context or setting in which it is implemented**	Not outline	Unclear/Unable to assess	
**9. The degree to which the effects of the intervention are modified by recipient or provider factors**	Not outlined	Unclear/Unable to assess	

**10. The nature of the causal pathway between the intervention and the outcome it is intended to effect**	Not outlined	Unclear/Unable to assess	

Study ID: Cormack 1994
Study title: Evaluation of an easy, cost-effective strategy for cutting benzodiazepine use in general practice

**Study and Intervention details**			
Study aims/objectives	"to assess the effect of a letter from the general practitioner, suggesting a reduction in the use of benzodiazepines, and whether the impact of the letter could be increased by the addition of information on how to tackle drug reduction."		
Study outcome(s)	"to try to reduce or stop their benzodiazepine medication"		
Details of intervention/s and the comparison (including usual care)	**Intervention Group 1 :**<br>"received a letter from their general practitioner asking them to try to reduce or stop their benzodiazepine medication and advising that this should be done gradually (Appendix 1)"<br>**Intervention Group 2:**<br>"received the same letter, followed at monthly intervals by four information sheets giving advice about reducing medication, including practical suggestions for coping without drugs."<br>**Control Group:**<br>"received no intervention"		
Intervention aim/objectives	"to try to reduce or stop their benzodiazepine medication"		
Intervention deliverer	GPs – "received a letter from their general practitioner asking them to try to reduce or stop their benzodiazepine medication and advising that this should be done gradually"		
Intervention target/recipient (which may include individuals, groups of individuals and other entities)	"Long-term users were identified by general practitioners and divided into three groups: two intervention groups and a control group"		
Target behaviour and component actions (i.e. whose behaviour the intervention intended to change and what were the component actions involved in that behaviour)	Not much detail given. The target behaviour was "reduction of benzodiazepine use"		
**Intervention Group 1:**			
**Core dimension**	**Description of the intervention in the review**	**Judgement**	**Support for judgement**
**1. Organisational levels and categories targeted by the intervention**	"long-term users of benzodiazepines in general practice were divided into three groups: two intervention groups and a control group."	Single category	Only patients being targeted by intervention.
**2. Behaviour or actions of intervention recipients or participants to which the intervention is directed**	"reduction of benzodiazepine use"	Single target	Only one behaviour targeted – reduction in benzodiazepine consumption
**3. Active components included in the intervention, in relation to the comparison**	"The first intervention group received a letter	One component	Only one component to

	from their general practitioner asking that benzodiazepine use be gradually reduced and perhaps, in time, stopped.”		the intervention – a letter from the patients GP
**4. The degree of tailoring intended or flexibility permitted across sites or individuals in applying or implementing the intervention**	“Those in intervention group one received a letter from their general practitioner asking them to try to reduce or stop their benzodiazepine medication and advising that this should be done gradually (Appendix 1).”	Inflexible	Same letter was given to all the patients – no tailoring.
**5. The level of skill required by those delivering the intervention in order to meet the intervention’s objectives**	Not explicitly mentioned in text –“received a letter from their general practitioner asking that benzodiazepine use be gradually reduced and perhaps, in time, stopped” Doctors likely had to support patients in reducing benzodiazepine consumption. .	Intermediate level skills	Intermediate level skills required to support BENZODIAZEPINE withdrawal.
**6. The level of skill required for the targeted behaviour when entering the included studies by those receiving the intervention, in order to meet the intervention’s objectives**	Not explicitly stated in the report – high level skills required to undergo successful benzodiazepine discontinuation	High level skills	High level skill to undergo benzodiazepine discontinuation successfully.
**Optional dimension**	**Description of the intervention in the review**	**Judgement**	**Support for judgement**
**7. The degree of interaction between intervention components, including the independence / interdependence of intervention components**	Not outlined	Unclear/Unable to assess	
**8. The degree to which the effects of the intervention are dependent on the context or setting in which it is implemented**	Not outline	Unclear/Unable to assess	
**9. The degree to which the effects of the intervention are modified by recipient or provider factors**	Not outlined	Unclear/Unable to assess	
**10. The nature of the causal pathway between the intervention and the outcome it is intended to effect**	Not outlined	Unclear/Unable to assess	
**Intervention Group 2**			
**Core dimension**	**Description of the intervention in the review**	**Judgement**	**Support for judgement**

**1. Organisational levels and categories targeted by the intervention**	"long-term users of benzodiazepines in general practice were divided into three groups: two intervention groups and a control group."	Single category	Only patients being targeted by intervention.
**2. Behaviour or actions of intervention recipients or participants to which the intervention is directed**	"reduction of benzodiazepine use"	Single target	Only one behaviour targeted – reduction in benzodiazepine consumption
**3. Active components included in the intervention, in relation to the comparison**	Intervention group two received the same letter, followed at monthly intervals by four information sheets giving advice about reducing medication, including practical suggestions for coping without drugs.	More than one component and delivered as a bundle	The letter preceded the information sheets – therefore components were delivered as a bundle.
**4. The degree of tailoring intended or flexibility permitted across sites or individuals in applying or implementing the intervention**	"Intervention group two received the same letter, followed at monthly intervals by four information sheets giving advice about reducing medication, including practical suggestions for coping without drugs"	Inflexible	Same letter and information sheets received by all patients – no tailoring of intervention.
**5. The level of skill required by those delivering the intervention in order to meet the intervention's objectives**	Not explicitly mentioned in text.<br>Doctors likely had to support patients in reducing benzodiazepine consumption. .	Intermediate level skills	Intermediate level skills required to support BENZODIAZEPINE withdrawal.
**6. The level of skill required for the targeted behaviour when entering the included studies by those receiving the intervention, in order to meet the intervention's objectives**	Not explicitly stated in the report – high level skills required to undergo successful benzodiazepine discontinuation	High level skills	High level skill to undergo benzodiazepine discontinuation successfully.
**Optional dimension**	**Description of the intervention in the review**	**Judgement**	**Support for judgement**
**7. The degree of interaction between intervention components, including the independence / interdependence of intervention components**	Not outlined	Unclear/Unable to assess	
**8. The degree to which the effects of the intervention are dependent on the context or setting in which it is implemented**	Not outline	Unclear/Unable to assess	

**9. The degree to which the effects of the intervention are modified by recipient or provider factors**	Not outlined	Unclear/Unable to assess	
**10. The nature of the causal pathway between the intervention and the outcome it is intended to effect**	Not outlined	Unclear/Unable to assess	

Study ID: Vicens 2006

Study title: Withdrawal from long-term benzodiazepine use: randomised trial in family practice

Study and Intervention details			
Study aims/objectives	"to appraise the efficacy of a structured intervention in primary care, consisting of a brief standardised advice and a stepwise dose reduction, for discontinuing benzodiazepine use"		
Study outcome(s)	"The main efficacy variable was benzodiazepine use at 12 months, although data was also taken at 6 months"		
Details of intervention/s and the comparison (including usual care)	"The intervention consisted of an interview with a standardised message that had been developed previously through a qualitative study on four focal groups, each one with eight to 12 chronic consumers of benzodiazepines (one group of men and three of women, grouped per age). The content of the message is given in Box 1. Patients in the intervention group underwent a gradual reduction of benzodiazepine dose, with control visits every 15 days. The dose was reduced between 10 and 25% of the initial dose fortnightly. For patients in the control group, the same information was taken on personal, clinical, benzodiazepine use and psychological tests. They did not receive the structured intervention, being managed according to usual practice, and informed of the convenience of reducing the use of benzodiazepines"		
Intervention aim/objectives	"withdrawal of long-term benzodiazepine use"		
Intervention deliverer	Physicians - "Thirteen family physicians from three primary care centres took part in the trial."		
Intervention target/recipient (which may include individuals, groups of individuals and other entities)	"patients visiting the collaborating physicians, those aged 14–75 years and who were taking benzodiazepines at least five times a week for over a year,"		
Target behaviour and component actions (i.e. whose behaviour the intervention intended to change and what were the component actions involved in that behaviour)	"withdrawal of long-term benzodiazepine use"		
**Core dimension**	**Description of the intervention in the review**	**Judgement**	**Support for judgement**
**1. Organisational levels and categories targeted by the intervention**	"patients visiting the collaborating physicians, those aged 14–75 years and who were taking benzodiazepines at least five times a week for over a year,"	Single category	Intervention was only targeted at patients.
**2. Behaviour or actions of intervention recipients or participants to which the intervention is directed**	"withdrawal of long-term benzodiazepine use"	Single target	Only one behaviour targeted by the intervention – benzodiazepine use.
**3. Active components included in the intervention, in relation to the comparison**	"intervention consisted of an interview with a	More than one component and	The intervention

	standardised message that had been developed previously through a qualitative study on four focal groups, each one with eight to 12 chronic consumers of benzodiazepines (one group of men and three of women, grouped per age). The content of the message is given in Box 1. Patients in the intervention group underwent a gradual reduction of benzodiazepine dose, with control visits every 15 days. The dose was reduced between 10 and 25% of the initial dose fortnightly.”	delivered as a bundle	consisted of a number of components delivered as a bundle – components performed in a series of steps.
**4. The degree of tailoring intended or flexibility permitted across sites or individuals in applying or implementing the intervention**	“The intervention consisted of an interview with a standardised message that had been developed previously through a qualitative study on four focal groups, each one with eight to 12 chronic consumers of benzodiazepines (one group of men and three of women, grouped per age). The content of the message is given in Box 1. Patients in the intervention group underwent a gradual reduction of benzodiazepine dose, with control visits every 15 days.”	Moderately tailored/flexible	While the interviews consisted of a standardised message, it is likely that the consultation would have been tailored towards each patient and there would have been some flexibility in the dosing schedule.
**5. The level of skill required by those delivering the intervention in order to meet the intervention’s objectives**	Not explicitly mentioned – intermediate skills required to promote and support benzodiazepine withdrawal	Intermediate level skills	intermediate skills required to promote and support benzodiazepine withdrawal

**6. The level of skill required for the targeted behaviour when entering the included studies by those receiving the intervention, in order to meet the intervention's objectives**	Not explicitly stated in the report – high level skills required to undergo successful benzodiazepine discontinuation	High level skills	High level skill to undergo benzodiazepine discontinuation successfully.
**Optional dimension**	**Description of the intervention in the review**	**Judgement**	**Support for judgement**
**7. The degree of interaction between intervention components, including the independence / interdependence of intervention components**	Not outlined	Unclear/Unable to assess	
**8. The degree to which the effects of the intervention are dependent on the context or setting in which it is implemented**	Not outline	Unclear/Unable to assess	
**9. The degree to which the effects of the intervention are modified by recipient or provider factors**	Not outlined	Unclear/Unable to assess	
**10. The nature of the causal pathway between the intervention and the outcome it is intended to effect**	Not outlined	Unclear/Unable to assess	

Study ID: Kuntz 2019
Study title: Patient Education and Pharmacist Consultation Influence on Nonbenzodiazepine Sedative Medication Deprescribing Success for Older Adults

**Study and Intervention details**	
Study aims/objectives	"evaluated the impact of direct-to-patient education, with or without a pharmacist consultation, on Z-drug discontinuation among Kaiser Permanente Northwest members age 64 years and older."
Study outcome(s)	"The primary study outcome was discontinuation of Z-drugs during 6-month follow-up, defined as a patient not receiving a Z-drug dispensing from a KPNW pharmacy during that time."<br>"secondary outcomes, which included hospitalization, outpatient face-to-face encounters, and urgent care and Emergency Department visits during the 6-month follow-up. We also examined the number of Z-drug dispensing during follow-up for patients who did not discontinue use."
Details of intervention/s and the comparison (including usual care)	**Group 1: Education Only**<br>"received a letter from their prescribing physician, an educational brochure, and a quiz. Educational materials were developed by a team of primary care and geriatric health care physicians, pharmacists, and researchers. Prescriber letter text explained the reason for the letter and encouraged patients to reconsider their Z-drug use. The brochure presented evidence of Z-drug-induced harms, suggestions for effective pharmacologic and nonpharmacologic alternatives to treat insomnia, and a visual tapering schedule with further instructions. The quiz reiterated messages in the educational brochure by providing a self-assessment about Z-drug use risks"<br>**Group 2: Education and Arms**<br>As above +<br>"A pharmacist called patients in the Ed+ study arm 2 to 4 weeks after they received the educational materials. During these telephone consultations, the pharmacist would discuss and reinforce information in the educational mailing; assess patient barriers to Z-drug discontinuation; provide personalized guidance on tapering, recommendations for care coordination opportunities available through specialty departments such as sleep medicine, mental health, and addiction medicine; and answer questions. This format also provided the opportunity to discuss Z-drug alternatives, including sleep hygiene techniques and safer medications. The pharmacist had prescriber approval and a protocol that allowed for a switch to safer sleep medications"
Intervention aim/objectives	Z-drug discontinuation
Intervention deliverer	Group 1: GP<br>Group 2: Pharmacists and GPs<br>"A pharmacist called patients in the Ed+ study arm 2 to 4 weeks after they received the educational materials."

	"received a letter from their prescribing physician"
Intervention target/recipient (which may include individuals, groups of individuals and other entities)	"Patients were eligible for the deprescribing intervention if they were at least age 64 years and received 2 or 3 prescription medication dispensing's of a Z-drug—including eszopiclone, zolpidem, or zaleplon—during 2016"
Target behaviour and component actions (i.e. whose behaviour the intervention intended to change and what were the component actions involved in that behaviour)	Z-drug use<br>"engage them in shared decision making regarding discontinuation"

**Ed Group**			
**Core dimension**	**Description of the intervention in the review**	**Judgement**	**Support for judgement**
**1. Organisational levels and categories targeted by the intervention**	"Patients were eligible for the deprescribing intervention if they were at least age 64 years and received 2 or 3 prescription medication dispensing of a Z-drug—including eszopiclone, zolpidem, or zaleplon—during 2016"	Single category	Only patients are targeted by the intervention.
**2. Behaviour or actions of intervention recipients or participants to which the intervention is directed**	"engage them in shared decision making regarding discontinuation"	Single target	One behaviour targeted – z-drug discontinuation
**3. Active components included in the intervention, in relation to the comparison**	"received a letter from their prescribing physician, an educational brochure, and a quiz. Educational materials were developed by a team of primary care and geriatric health care physicians, pharmacists, and researchers. Prescriber letter text explained the reason for the letter and encouraged patients to reconsider their Z-drug use. The brochure presented evidence of Z-drug-induced harms, suggestions for effective pharmacologic and nonpharmacologic alternatives to treat insomnia, and a visual tapering schedule with further instructions. The	More than one component.	Intervention was delivered as a package consisting of a letter, a brochure, and a quiz. No specific order in which intervention components were to be carried out.

	quiz reiterated messages in the educational brochure by providing a self-assessment about Z-drug use risks”		
**4. The degree of tailoring intended or flexibility permitted across sites or individuals in applying or implementing the intervention**	“received a letter from their prescribing physician, an educational brochure, and a quiz. Educational materials were developed by a team of primary care and geriatric health care physicians, pharmacists, and researchers. Prescriber letter text explained the reason for the letter and encouraged patients to reconsider their Z-drug use. The brochure presented evidence of Z-drug-induced harms, suggestions for effective pharmacologic and nonpharmacologic alternatives to treat insomnia, and a visual tapering schedule with further instructions. The quiz reiterated messages in the educational brochure by providing a self-assessment about z-drug use risks”	Inflexible	No tailoring of intervention to different patients
**5. The level of skill required by those delivering the intervention in order to meet the intervention’s objectives**	Not explicitly mentioned – intermediate skills required to promote and support benzodiazepine withdrawal	Intermediate level skills	Intermediate skills required to promote and support benzodiazepine withdrawal
**6. The level of skill required for the targeted behaviour when entering the included studies by those receiving the intervention, in order to meet the intervention’s objectives**	Not explicitly stated in the report – high level skills required to undergo successful benzodiazepine discontinuation	High level skills	High level skill to undergo benzodiazepine discontinuation successfully.
**Optional dimension**	**Description of the intervention in the review**	**Judgement**	**Support for judgement**
**7. The degree of interaction between intervention components, including the**	Not outlined	Unclear/Unable to assess	

independence / interdependence of intervention components			
8. The degree to which the effects of the intervention are dependent on the context or setting in which it is implemented	Not outline	Unclear/Unable to assess	
9. The degree to which the effects of the intervention are modified by recipient or provider factors	Not outlined	Unclear/Unable to assess	
10. The nature of the causal pathway between the intervention and the outcome it is intended to effect	Not outlined	Unclear/Unable to assess	

Ed + Arms Group			
**Core dimension**	**Description of the intervention in the review**	**Judgement**	**Support for judgement**
**1. Organisational levels and categories targeted by the intervention**	"Patients were eligible for the deprescribing intervention if they were at least age 64 years and received 2 or 3 prescription medication dispensings of a Z-drug—including eszopiclone, zolpidem, or zaleplon—during 2016"	Single category	
**2. Behaviour or actions of intervention recipients or participants to which the intervention is directed**	"engage them in shared decision making regarding discontinuation"	Single target	One behaviour targeted – z-drug discontinuation
**3. Active components included in the intervention, in relation to the comparison**	"received a letter from their prescribing physician, an educational brochure, and a quiz. Educational materials were developed by a team of primary care and geriatric health care physicians, pharmacists, and researchers. Prescriber letter text explained the reason for the letter and encouraged patients to reconsider their Z-drug use. The brochure presented evidence of Z-drug-induced harms, suggestions for effective pharmacologic and nonpharmacologic alternatives to treat	More than one component delivered a bundle	Intervention consisted of multiple components – letter, brochure quiz followed by a call from the pharmacist 2-4 weeks later. Letter and information preceded call from pharmacist.

	insomnia, and a visual tapering schedule with further instructions. The quiz reiterated messages in the educational brochure by providing a self-assessment about Z-drug use risks"<br>"A pharmacist called patients in the Ed+ study arm 2 to 4 weeks after they received the educational materials. During these telephone consultations, the pharmacist would discuss and reinforce information in the educational mailing; assess patient barriers to Z-drug discontinuation; provide personalized guidance on tapering, recommendations for care coordination opportunities available through specialty departments such as sleep medicine, mental health, and addiction medicine; and answer questions. This format also provided the opportunity to discuss Z-drug alternatives, including sleep hygiene techniques and safer medications. The pharmacist had prescriber approval and a protocol that allowed for a switch to safer sleep medications"		
**4. The degree of tailoring intended or flexibility permitted across sites or individuals in applying or implementing the intervention**	"During these telephone consultations, the pharmacist would discuss and reinforce information in the educational mailing; assess patient barriers to Z-drug discontinuation; provide personalized guidance on tapering,		Guidance of tapering was tailored to the patient's needs – some degree of flexibility.

	recommendations for care coordination opportunities available through specialty departments such as sleep medicine, mental health, and addiction medicine; and answer questions. This format also provided the opportunity to discuss Z-drug alternatives, including sleep hygiene techniques and safer medications. The pharmacist had prescriber approval and a protocol that allowed for a switch to safer sleep medications”	Moderately flexible	
**5. The level of skill required by those delivering the intervention in order to meet the intervention’s objectives**	Not explicitly mentioned – intermediate skills required to promote and support benzodiazepine withdrawal	Intermediate level skills	intermediate skills required to promote and support benzodiazepine withdrawal
**6. The level of skill required for the targeted behaviour when entering the included studies by those receiving the intervention, in order to meet the intervention’s objectives**	Not explicitly stated in the report – high level skills required to undergo successful benzodiazepine discontinuation	High level skills	High level skill to undergo benzodiazepine discontinuation successfully.
**Optional dimension**	**Description of the intervention in the review**	**Judgement**	**Support for judgement**
**7. The degree of interaction between intervention components, including the independence / interdependence of intervention components**	Not outlined	Unclear/Unable to assess	
**8. The degree to which the effects of the intervention are dependent on the context or setting in which it is implemented**	Not outline	Unclear/Unable to assess	
**9. The degree to which the effects of the intervention are modified by recipient or provider factors**	Not outlined	Unclear/Unable to assess	
**10. The nature of the causal pathway between the intervention and the outcome it is intended to effect**	Not outlined	Unclear/Unable to assess	

Study ID: Navy 2018

Study title: Clinical Pharmacist Intervention to Engage Older Adults in Reducing Use of Alprazolam

**Study and Intervention details**	
Study aims/objectives	"to assess whether an educational letter explaining the risks of alprazolam can provide an impetus for older adults to call a clinical pharmacist to participate more actively in their own health care and engage in benzodiazepine use reduction"
Study outcome(s)	"the primary outcome was a comparison between the intervention and control groups on the composite rate of patients who 1) had no alprazolam dispensing at any time during the six month follow up, 2) had an alprazolam dose reduction at any time during the six-month follow-up, or 3) interchanged to an alternate medication at any time during the six-month follow up"<br>"secondary outcomes included comparisons between the groups on the individual outcomes of the composite and rate of intervention patients who called the study CP within 14 days of the study letter being mailed"
Details of intervention/s and the comparison (including usual care)	"An intervention letter |(appendix) was developed with input from the KPCO geriatric CPs and the medical director of geriatrics and Medicare. The letter was addressed to the patient and outlined: 1) the reason for it being sent (i.e., the patient was prescribed alprazolam), 2) that there are risks to taking alprazolam, 3) organisations that recommend against taking alprazolam, 4) alprazolam's side effects, 5) possible alternate treatment options, 6) a request to call the study CP to discuss treatment options, 7) not to stop taking alprazolam without talking to the study CP, and 8) the telephone number and times to call the study"<br>"letters were sent via the US postal service on a rolling basis to allow the study CP to manage telephone calls and use-reduction efforts, if a patient called the study CP, usual care was provided. During the usual care discussion, the study CP assessed the patients decisional capacity to understand and follow instructions. If the patient could not comprehend the information provided or make a reasoned choice regarding alprazolam dose reduction or discontinuation, the study CP did not proceed with study…"<br>"for patients who agreed to participate, alternate treatment options were discussed on a case-by-case basis…"<br>"if the patient was agreeable, the study CP collaborated with the patient's primary care provider (PCP) to develop an individualised alprazolam taper plan. The study CP would monitor the patient for withdrawal symptoms by telephone follow-up throughout the duration of the taper. For patients who did not agree to study participation, usual care was provided"
Intervention aim/objectives	"provide an impetus for older adults to call a clinical pharmacist to participate more actively in their own health care and engage in benzodiazepine use reduction"
Intervention deliverer	Clinical pharmacist

Intervention target/recipient (which may include individuals, groups of individuals and other entities)	"patients were included if they were 65 years of age or older as of December 15 2016, continuous members of KPCO for the 3 months prior, members from the Denver/ Boulder metropolitan area who resided at home, had a current supply of alprazolam as of December 15, 2017, and had four outpatient dispensings of alprazolam during the previous 12 months with a total of at least 90 days of medication supplied"		
Target behaviour and component actions (i.e. whose behaviour the intervention intended to change and what were the component actions involved in that behaviour)	"provide an impetus for older adults to call a clinical pharmacist to participate more actively in their own health care and engage in benzodiazepine use reduction"		
**Core dimension**	**Description of the intervention in the review**	**Judgement**	**Support for judgement**
**1. Organisational levels and categories targeted by the intervention**	"patients were identified for study participation from pharmacy dispensing records"	Single category	Intervention only targets patients
**2. Behaviour or actions of intervention recipients or participants to which the intervention is directed**	"provide an impetus for older adults to call a clinical pharmacist to participate more actively in their own health care and engage in benzodiazepine use reduction"	Single target	Only one behaviour targeted – benzodiazepine use.
**3. Active components included in the intervention, in relation to the comparison**	"An intervention letter |(appendix) was developed with input from the KPCO geriatric CPs and the medical director of geriatrics and Medicare. The letter was addressed to the patient and outlined: 1) the reason for it being sent (i.e., the patient was prescribed alprazolam), 2) that there are risks to taking alprazolam, 3) organisations that recommend against taking alprazolam, 4) alprazolam's side effects, 5) possible alternate treatment options, 6) a request to call the study CP to discuss treatment options, 7) not to stop taking alprazolam without talking to the study CP, and 8) the telephone	More than one component and delivered as a bundle	The first component of the intervention was a letter. If the patient called the clinical pharmacist and was eligible the next component was to provide a tapering plan to reduce the patients dose of alprazolam.

	number and times to call the study"<br>"letters were sent via the US postal service on a rolling basis to allow the study CP to manage telephone calls and use-reduction efforts, if a patient called the study CP, usual care was provided. During the usual care discussion, the study CP assessed the patients decisional capacity to understand and follow instructions. If the patient could not comprehend the information provided or make a reasoned choice regarding alprazolam dose reduction or discontinuation, the study CP did not proceed with study…"<br>"for patients who agreed to participate, alternate treatment options were discussed on a case-by-case basis…"<br>"if the patient was agreeable, the study CP collaborated with the patient's primary care provider (PCP) to develop an individualised alprazolam taper plan. The study CP would monitor the patient for withdrawal symptoms by telephone follow-up throughout the duration of the taper. For patients who did not agree to study participation, usual care was provided"		
**4. The degree of tailoring intended or flexibility permitted across sites or individuals in applying or implementing the intervention**	"for patients who agreed to participate, alternate treatment options were discussed on a case-by-case basis…"	Moderately tailored/flexible	Intervention was tailored to specific patients with individualised tapering plans.

	"if the patient was agreeable, the study CP collaborated with the patient's primary care provider (PCP) to develop an individualised alprazolam taper plan. The study CP would monitor the patient for withdrawal symptoms by telephone follow-up throughout the duration of the taper.		
**5. The level of skill required by those delivering the intervention in order to meet the intervention's objectives**	Not explicitly stated – intermediate skills required to promote and support benzodiazepine withdrawal	Intermediate level skills	intermediate skills required to promote and support BENZODIAZEPI NE withdrawal
**6. The level of skill required for the targeted behaviour when entering the included studies by those receiving the intervention, in order to meet the intervention's objectives**	Not explicitly stated in the report – high level skills required to undergo successful benzodiazepine discontinuation	High level skills	High level skill to undergo benzodiazepine discontinuation successfully.
**Optional dimension**	**Description of the intervention in the review**	**Judgement**	**Support for judgement**
**7. The degree of interaction between intervention components, including the independence / interdependence of intervention components**	Not outlined	Unclear/Unable to assess	
**8. The degree to which the effects of the intervention are dependent on the context or setting in which it is implemented**	Not outline	Unclear/Unable to assess	
**9. The degree to which the effects of the intervention are modified by recipient or provider factors**	Not outlined	Unclear/Unable to assess	
**10. The nature of the causal pathway between the intervention and the outcome it is intended to effect**	Not outlined	Unclear/Unable to assess	

Study ID: Vicens 2014
Study title: Comparative efficacy of two interventions to discontinue long-term benzodiazepine use: cluster randomised controlled trial in primary care

**Study and Intervention details**	
Study aims/objectives	"This study assessed two interventions: a structured educational intervention with gradual tapering backed up by fortnightly follow-up visits (SIF) and the same structured educational intervention supported by written instruction rather than follow-up visits (SIW), requiring less GP involvement.<br>The aim was to compare the effectiveness of these two interventions with that of usual care on the discontinuation of long-term benzodiazepine use in primary care patients, delivered at the level of the GP. We also attempted to determine the effectiveness of each intervention relative to patient characteristics."
Study outcome(s)	"The primary outcome was benzodiazepine discontinuation at 12 months, assessed in a personal interview and defined as self-declared non-consumption or consumption of fewer than four doses in the previous month. Consumption was reviewed and confirmed by prescription claims in the clinical records."<br>"Secondary outcomes were benzodiazepine discontinuation at 6 months and safety outcomes measured at 6 months and 12 months, including changes in anxiety and depression symptoms, changes in sleep satisfaction, alcohol consumption and withdrawal symptoms."
Details of intervention/s and the comparison (including usual care)	**Both groups:**<br>"Practitioners assigned to the SIF and SIW groups attended a supplementary 3 h workshop on structured interviews, individualised patient information and training in managing benzodiazepine discontinuation and optimal gradual dose reduction. In addition, GPs assigned to the SIF group attended a brief 30 min workshop to standardise the dose-reduction follow-up visits. Training was provided by researchers with extensive experience in the management of benzodiazepine withdrawal.<br>The SIF and SIW interventions were both based on a structured educational interview and GP-tailored stepped benzodiazepine dose reduction. These two interventions differed only in the follow-up"<br>**SIF:**<br>"The content of the educational interview was structured and included four key points: (a) information on benzodiazepine dependence, abstinence and withdrawal symptoms; (b) the risks of long-term use, memory and cognitive impairment, accidents and falls; (c) reassurance about reducing medication; (d) a self-help leaflet to improve sleep quality if patients were taking benzodiazepines for insomnia<br>Practitioners assigned to the SIF and SIW groups attended a supplementary 3 h workshop on structured interviews, individualised patient information and training in managing benzodiazepine discontinuation and optimal gradual dose

	reduction. In addition, GPs assigned to the SIF group attended a brief 30 min workshop to standardise the dose-reduction follow-up visits. Training was provided by researchers with extensive experience in the management of benzodiazepine withdrawal. The SIF and SIW interventions were both based on a structured educational interview and GP-tailored stepped benzodiazepine dose reduction. These two interventions differed only in the follow-up. The content of the educational interview was structured and included four key points: (a) information on benzodiazepine dependence, abstinence and withdrawal symptoms; (b) the risks of long-term use, memory and cognitive impairment, accidents and falls; (c) reassurance about reducing medication; (d) a self-help leaflet to improve sleep quality if patients were taking benzodiazepines for insomnia"<br>After the first intervention visit patients in the SIF group were scheduled for follow-up appointments with their GPs every 2–3 weeks until the end of the dose reduction. The GPs reinforced education, reassured patients regarding withdrawal symptoms and obtained patient agreement for the next step in dose reduction."<br>**SIW:**<br>"Patients in the SIW group received written instructions reinforcing educational information at their first and only contact with their GP, along with a tailored gradual dose reduction until benzodiazepine cessation. No follow-up visit was scheduled, although patients could spontaneously request an appointment with their GP when needed."<br>**Control: "**Patients allocated to the control group received routine care; their GPs could provide brief advice but did not receive any specific recommendation about the management of long-term benzodiazepine use from the study trainers."
Intervention aim/objectives	"self-declared benzodiazepine discontinuation confirmed by prescription claims at 12 months"
Intervention deliverer	GPs – "Participating GPs were selected from 21 primary care centres in the three regions and were included if they were able to commit to taking part until completion"
Intervention target/recipient (which may include individuals, groups of individuals and other entities)	Patients eligible for the trial were aged 18–80 years and had been taking benzodiazepines daily for at least 6 months. Exclusion criteria were psychotic or personality disorder, or current treatment by a psychiatrist; severe anxiety, depressive disorder or severe medical illness including dementia and epilepsy as clinically assessed by the GP, or in cases where they considered that stopping benzodiazepine might be harmful; alcohol or illicit drug misuse; patient in residential care or terminally ill; inability to read and speak Spanish; or unwillingness to provide informed consent
Target behaviour and component actions (i.e. whose behaviour the intervention intended to change and what were the component actions involved in that behaviour)	"benzodiazepine discontinuation at 12 months"
**SIF Group**	

Core dimension	Description of the intervention in the review	Judgement	Support for judgement
**1. Organisational levels and categories targeted by the intervention**	"Patients eligible for the trial were aged 18–80 years and had been taking benzodiazepines daily for at least 6 months."	Single category	Intervention only targeted patients.
**2. Behaviour or actions of intervention recipients or participants to which the intervention is directed**	""benzodiazepine dose reduction"	Single target	Only one behaviour being targeted – benzodiazepine use.
**3. Active components included in the intervention, in relation to the comparison**	Practitioners assigned to the SIF and SIW groups attended a supplementary 3 h workshop on structured interviews, individualised patient information and training in managing benzodiazepine discontinuation and optimal gradual dose reduction. In addition, GPs assigned to the SIF group attended a brief 30 min workshop to standardise the dose-reduction follow-up visits. Training was provided by researchers with extensive experience in the management of benzodiazepine withdrawal. The SIF and SIW interventions were both based on a structured educational interview and GP-tailored stepped benzodiazepine dose reduction. These two interventions differed only in the follow-up<br>"The content of the educational interview was structured and included four key points: (a) information on benzodiazepine dependence, abstinence and withdrawal symptoms; (b) the risks of	More than one component and delivered as a bundle	Intervention consisted of a number of components which were delivered a bundle as there was a specific order.

	long-term use, memory and cognitive impairment, accidents and falls; (c) reassurance about reducing medication; (d) a self-help leaflet to improve sleep quality if patients were taking benzodiazepines for insomnia<br>After the first intervention visit patients in the SIF group were scheduled for follow-up appointments with their GPs every 2–3 weeks until the end of the dose reduction. The GPs reinforced education, reassured patients regarding withdrawal symptoms and obtained patient agreement for the next step in dose reduction.		
**4. The degree of tailoring intended or flexibility permitted across sites or individuals in applying or implementing the intervention**	Practitioners assigned to the SIF and SIW groups attended a supplementary 3 h workshop on structured interviews, individualised patient information and training in managing benzodiazepine discontinuation and optimal gradual dose reduction	Moderately tailored/flexible	Interviews were tailored based on patient information
**5. The level of skill required by those delivering the intervention in order to meet the intervention's objectives**	"General practitioners assigned to the three groups attended an hour-long workshop explaining the study protocol and providing training in filling out the case report form. Practitioners assigned to the SIF and SIW groups attended a supplementary 3 h workshop on structured interviews, individualised patient information and training in managing benzodiazepine discontinuation and	Intermediate level skills	GPs had to undergo a number of training sessions in order to develop the skills to advise patients on benzodiazepine discontinuation and to carry out the intervention.

	optimal gradual dose reduction. In addition, GPs assigned to the SIF group attended a brief 30 min workshop to standardise the dose-reduction follow-up visits. Training was provided by researchers with extensive experience in the management of benzodiazepine withdrawal"		
6. **The level of skill required for the targeted behaviour when entering the included studies by those receiving the intervention, in order to meet the intervention's objectives**	Not explicitly stated in the report – high level skills required to undergo successful benzodiazepine discontinuation	High level skills	High level skill to undergo benzodiazepine discontinuation successfully.
**Optional dimension**	**Description of the intervention in the review**	**Judgement**	**Support for judgement**
7. **The degree of interaction between intervention components, including the independence / interdependence of intervention components**	Not outlined	Unclear/Unable to assess	
8. **The degree to which the effects of the intervention are dependent on the context or setting in which it is implemented**	Not outline	Unclear/Unable to assess	
9. **The degree to which the effects of the intervention are modified by recipient or provider factors**	"Withdrawal at 12 months did not differ by gender, age, short or long half-depression (HADS score), insomnia (Oviedo questionnaire) or degree of dependence (SDS). benzodiazepine use,"	Largely independent of individual-level factors<br>–	Effect of intervention was not impacted by patient characteristics.
10. **The nature of the causal pathway between the intervention and the outcome it is intended to effect**	Not outlined	– Unclear/Unable to assess	

**SIW Group**			
**Core dimension**	**Description of the intervention in the review**	**Judgement**	**Support for judgement**
1) **Organisational levels and categories targeted by the intervention**	"Patients eligible for the trial were aged 18–80 years and had been taking benzodiazepines daily for at least 6 months."	Single category	Intervention only targeted patients.
2) **Behaviour or actions of intervention recipients or participants to which the intervention is directed**	""benzodiazepine dose reduction"	Single target	Only one behaviour being targeted

			– benzodiazepine use.
**3) Active components included in the intervention, in relation to the comparison**	"Practitioners assigned to the SIF and SIW groups attended a supplementary 3 h workshop on structured interviews, individualised patient information and training in managing benzodiazepine discontinuation and optimal gradual dose reduction. …The SIF and SIW interventions were both based on a structured educational interview and GP-tailored stepped benzodiazepine dose reduction. These two interventions differed only in the follow-up.<br>"Patients in the SIW group received written instructions reinforcing educational information at their first and only contact with their GP, along with a tailored gradual dose reduction until benzodiazepine cessation. No follow-up visit was scheduled, although patients could spontaneously request an appointment with their GP when needed."	More than one component and delivered as a bundle	Intervention consists of a number of different components which were delivered in a specific order – interview with GP preceded written instructions.
**4) The degree of tailoring intended or flexibility permitted across sites or individuals in applying or implementing the intervention**	Practitioners assigned to the SIF and SIW groups attended a supplementary 3 h workshop on structured interviews, individualised patient information and training in managing benzodiazepine discontinuation and optimal gradual dose reduction	Moderately tailored/flexible	Interviews were tailored based on patient information
**5) The level of skill required by those delivering the intervention in order to meet the intervention's objectives**	"General practitioners assigned to the three groups attended an hour-	Intermediate level skills<br>–	GPs had to undergo a number of

	long workshop explaining the study protocol and providing training in filling out the case report form. Practitioners assigned to the SIF and SIW groups attended a supplementary 3 h workshop on structured interviews, individualised patient information and training in managing benzodiazepine discontinuation and optimal gradual dose reduction. In addition, GPs assigned to the SIF group attended a brief 30 min workshop to standardise the dose-reduction follow-up visits. Training was provided by researchers with extensive experience in the management of benzodiazepine withdrawal"		training sessions in order to develop the skills to advise patients on BENZODIAZEPINE discontinuation and to carry out the intervention.
**6) The level of skill required for the targeted behaviour when entering the included studies by those receiving the intervention, in order to meet the intervention's objectives**	Not explicitly stated in the report – high level skills required to undergo successful benzodiazepine discontinuation	High level skills	High level skill to undergo benzodiazepine discontinuation successfully.
**Optional dimension**	**Description of the intervention in the review**	**Judgement**	**Support for judgement**
**7) The degree of interaction between intervention components, including the independence / interdependence of intervention components**	Not outlined	Unclear/Unable to assess	
**8) The degree to which the effects of the intervention are dependent on the context or setting in which it is implemented**	Not outline	Unclear/Unable to assess	
**9) The degree to which the effects of the intervention are modified by recipient or provider factors**	"Withdrawal at 12 months did not differ by gender, age, short or long half-depression (HADS score), insomnia (Oviedo questionnaire) or degree of dependence (SDS). benzodiazepine use,"	Largely independent of individual-level factors	Effect of intervention was not impacted by patient characteristics.
**10) The nature of the causal pathway between the intervention and the outcome it is intended to effect**	Not outlined	Unclear/Unable to assess	
